# THE CORRELATION BETWEEN SHBG AND BLOOD LIPID AND GLUCOSE AMONG CHINESE MIDDLE-AGED AND OLDER ADULTS

**DOI:** 10.1093/geroni/igae098.3024

**Published:** 2024-12-31

**Authors:** Meng Cao, Yaping Jin, Huili Yang, Yuanbin Li, Minhui Liu, Chongmei Huang

**Affiliations:** Ningxia Medical University, Yinchuan, Ningxia, China; Ningxia Medical University, Yinchuan, Ningxia, China; Ningxia Medical University, Yinchuan, Ningxia, China (People’s Republic); Ningxia Medical University, Yinchuan, Ningxia, China; Ningxia Medical University, Yinchuan, Ningxia, China; Ningxia Medical University, Yinchuan, Ningxia, China

## Abstract

To investigate the correlation between sex hormone binding globulin (SHBG) of serum lipid and blood glucose in middle-aged and older adults (≥ 45 years old) We recruit participants in five urban communities in Ningxia province, using a cluster stratified sampling method. Data from 1681 participants were analysed, with an average age of 63.53 ± 9.5 years old. The results showed the average level of TC, HDL, LDL, and blood glucose were lower in men than in women (P=0.000,P=0.000,P=0.000,P=0.004),LDL-c and FBG levels increased with age in men, but SHBG level decreased with age in women (P< 0.05). The results of spearman correlation analysis showed a negative correlation between SHBG and FBG (r=-0.73 P< 0.01). There was a positive correlation between age (r= 0.054 P< 0.05) and HDL-C (r=0.049 P< 0.05) and a positive correlation between SHBG level and TC (r=0.218 P< 0.05) and TG (r=0.254 P< 0.05) in Chinese women aged 75 years and more. Multiple linear regression analysis with SHBG as the dependent variable and gender, age, TC, TG, HDL-C, LDL-C and BMI as independent variables did not find statistically significant associations. Logistic regression analysis showed that sex, age, weight, SHBG,FBG and SH were risk factors for dyslipidemia. However, there was no significant correlation between SHBG level and dyslipidemia.

